# Britannilactone 1-O-acetate induced ubiquitination of NLRP3 inflammasome through TRIM31 as a protective mechanism against reflux esophagitis-induced esophageal injury

**DOI:** 10.1186/s13020-024-00986-y

**Published:** 2024-08-30

**Authors:** Ju Liu, Yang Xiao, Qianfei Xu, Yunyan Xu, Manman Guo, Yun Hu, Yan Wang, Yi Wang

**Affiliations:** 1 Office of Science and Technology Administration, Kunshan Hospital of Traditional Chinese Medicine, Kunshan, China; 2 Nanjing University of Chinese Medicine, Nanjing, 210023 China; 3 Pharmaceutical Department, Kunshan Hospital of Traditional Chinese Medicine, Kunshan, China; 4 Department of Spleen, Stomach and Hepatobiliary, Kunshan Hospital of Traditional Chinese Medicine, Kunshan, China; 5 Preventive Treatment Department, Kunshan Hospital of Traditional Chinese Medicine, Kunshan, China

**Keywords:** TRIM31, NLRP3, Gastroesophageal reflux disease, Reflux esophagitis, Britannilactone 1-O-acetate

## Abstract

**Background:**

Reflux esophagitis (RE) is a disease in which inflammation of the esophageal mucosa owing to the reflux of gastric contents into the esophagus results in cytokine damage. Britannilactone 1-O-acetate (Brt) has anti-inflammatory effects, significantly inhibiting the activation of the NLRP3 inflammasome, leading to a decrease in inflammatory factors including IL-1 β, IL-6, and TNF-α. However, the mechanism underlying its protective effect against RE-induced esophageal injury remains unclear. In the present study, we investigated the protective mechanism of TRIM31 against NLRP3 ubiquitination-induced RE both in vivo and in vitro.

**Methods:**

A model of RE was established in vivo in rats by the method of “4.2 mm pyloric clamp + 2/3 fundoplication”. In vitro, the mod was constructed by using HET-1A (esophageal epithelial cells) and exposing the cells to acid, bile salts, and acidic bile salts. The 3-[4,5-dimethylthiazol-2-yl]-2,5 diphenyl tetrazolium bromide (MTT) assay was used to screen the concentration of administered drugs, and the viability of HET-1A cells in each group. HE staining was used to assess the degree of pathological damage in esophageal tissues. Toluidine blue staining was used to detect whether the protective function of the esophageal epithelial barrier was damaged and restored. The enzyme-linked immunosorbent assay (ELISA) was used to detect the levels of IL-1 β, IL-6, and TNF-α factors in serum. Immunohistochemistry (IHC) was used to detect the expression level of NLRP3 in esophageal tissues. The molecular docking and **Co-**immunoprecipitation assay (Co-IP assay) were used to detect the TRIM31 interacts with NLRP3. Western blotting detected the Claudin-4, Claudin-5, The G-protein-coupled receptor calcium-sensitive receptor (CaSR), NLRP3, TRIM31, ASC, C-Caspase1, and Caspase1 protein expression levels.

**Results:**

Brt could alleviate RE inflammatory responses by modulating serum levels of IL-1 β, IL-6, and TNF-α. It also activated the expression of NLRP3, ASC, Caspase 1, and C-Caspase-1 in HET-1A cells. Brt also attenuated TRIM31/NLRP3-induced pathological injury in rats with RE through a molecular mechanism consistent with the in vitro results.

**Conclusions:**

Brt promotes the ubiquitination of NLRP3 through TRIM31 and attenuates esophageal epithelial damage induced by RE caused by acidic bile salt exposure. This study provides valuable insights into the mechanism of action of Brt in the treatment of RE and highlights its promising application in the prevention of NLRP3 inflammatory vesicle-associated inflammatory pathological injury.

**Graphical Abstract:**

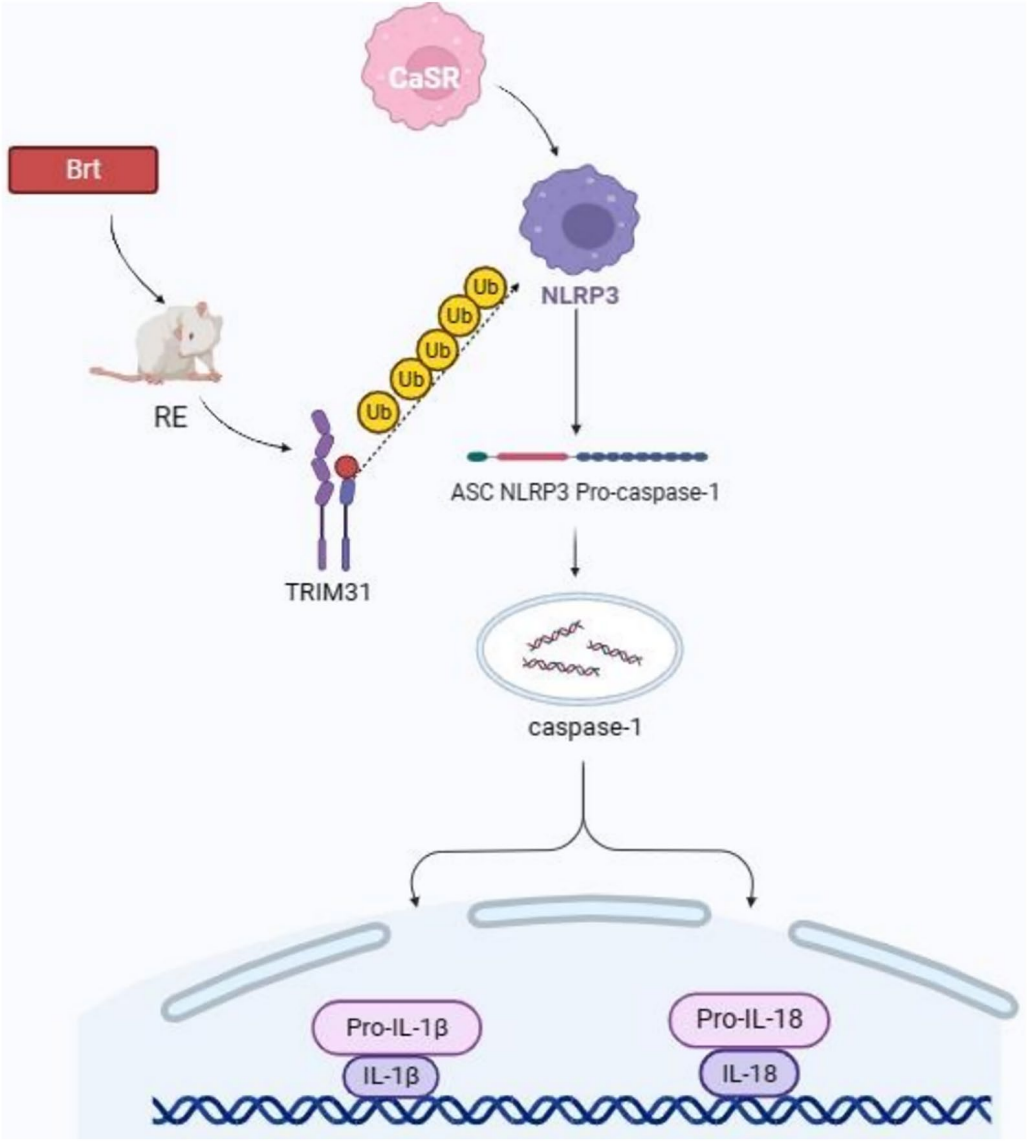

**Supplementary Information:**

The online version contains supplementary material available at 10.1186/s13020-024-00986-y.

## Introduction

Gastro-esophageal reflux disease (GERD) is one of the most common diseases, and it is estimated that approximately 20% of the adult population in the Western world has GERD [1]. The widespread prevalence of GERD imposes a huge economic burden on individuals and society [2]. Reflux esophagitis (RE) is a GERD subtype, with acid reflux, heartburn, vomiting, and dysphagia as the main clinical manifestations. The key link to RE is the recurrent chronic inflammation of the esophagus [3]. New theories of RE pathogenesis have suggested that it develops through cytokine-mediated injury rather than chemical injury and progresses to deeper layers of the epithelium [4]. The most common morphological abnormalities of the esophagus in RE are basal cell hyperplasia and connective tissue elongation caused by epithelial damage from acid/base exposure [5]. It has been shown that RE can lead to esophageal secretion of pro-inflammatory cytokines [6].

The NLRP3 inflammatory vesicle, comprising NLRP3, cysteine aspartate-specific protease-1 (Caspase-1), and ASC, is an essential component of the host's defense mechanism against pathogens [7–11]. NLRP3 expression is thought to be a limiting step in inflammatory vesicle activation [12, 13]. NLRP3 activation culminates in the formation of inflammatory vesicles containing ASC and procaspase-1 and this leads to cysteine asparaginase-1 activation [14], which is derived from its precursor protein, inflammatory cysteinyl asparaginase that hydrolyzes pro-IL-1β proteins to their mature, active form, in turn triggering inflammatory cell death, i.e. apoptosis [15]. NIMA-associated kinase (Nek7), a member of the mammalian NIMA-associated kinase (Neks) family, is an essential protein that functions downstream of potassium efflux to mediate NLRP3 inflammatory vesicle assembly and activation [16]. Major protein degradation pathways such as the ubiquitin–proteasome pathway, present in almost all human cells, offer specificity and control over the strength of the innate immune response [17, 18].

Tripartite motif (TRIM) family proteins promote the degradation of their respective substrates through the ubiquitin–proteasome pathway and are associated with the negative regulation of innate immunological reactions [19]. TRIM31 is a new member of the TRIM family that functions as an E3 ubiquitin ligase. It can promote substrate degradation through the ubiquitin–proteasome pathway and participates in various cell biological processes such as apoptosis, proliferation, and metabolism [20]. TRIM31 has been reported to negatively regulate the activity of NLRP3 inflammatory vesicles [21]. Some studies have shown that TRIM31 negatively regulates NLRP3 inflammasome activation in Helicobacter pylori (Hp)-associated gastritis by affecting ROS production and autophagy in gastric epithelial cells [22]. Overall, it is a key conjecture that the TRIM31/NLRP3 pathway causing inflammation is a further contributor to RE.

In traditional Chinese medicine (TCM), *Inula japonica Thunb.* is mainly used to treat of wind-cold cough, phlegm drink accumulation, chest and diaphragm plumpness, wheezing, and coughing with excessive phlegm.The flowers of the perennial herb *Inula japonica Thunb.* contain Britannilctone 1-O-acetate (Brt) as the main active ingredient with anti-inflammatory, hypoglycaemic, anti-bacterial, anti-cancer, and anti-hepatitis activities [23–27]. Brt is a sesquiterpene lactone enriched with *Inula japonica Thunb.* for the treatment of bronchitis and inflammation, and it has been reported that the cytokine inhibitory treatment is an effective treatment for numerous inflammatory illnesses [28]. However, the mechanism of action of Brt in RE is not yet known and further investigative experiments are required.

In this study, we describe the mechanism by which TRIM31 negatively regulates NLRP3 inflammasome activity. TRIM31 binds directly to NLRP3, which promotes NLRP3-linked polyubiquitination and proteasomal degradation. In line with this, TRIM31 deficiency promotes the activation of NLRP3 inflammasomes and enhances the secretion of IL-1 β, IL-16, and TNF-α, thereby exacerbating RE. By contrast, Brt treatment improved the general condition and pathological lesions of the esophageal epithelium in modified RE rats, reversed the harmful effects of acidic bile salts, downregulated the TRIM31-mediated NLRP3 inflammasome pathway, and reduced Caspase-1 activity, ASC release, and inflammatory factors. Therefore, TRIM31 may be a key factor in regulating NLRP3 vesicle activation. A series of studies have been conducted based on this hypothesis.

## Materials and methods

### Animal and drug management

This study was approved by the Committee on the Ethics of Animal Experiments of Nanjing University of Chinese Medicine (registration number of the ethical approval: A2308047). Male Sprague Dawley (SD) rats, (7 weeks old, 220 ± 20 g weight) from Hangzhou Medical College (SCXK (Zhe) 2019–0002), were maintained at 25 ± 1 ℃ and 65 ± 5% relative humidity, with a 12 h cycle for light and dark. Before the onset of the experiment, all rats were maintained with free available feed and water for 1 week to adapt, and they were housed in the animal house of the Nanjing University of Chinese Medicine (specific pathogen-free [SPF] grade). All mice were randomly divided into six groups: sham, mod, Brt-L (5 mg/kg), Btr-M (10 mg/kg), Brt-H (20 mg/kg), and OME (omeprazole 4.17 mg/kg). Brt (Catalog No: B21408) was purchased from Shanghai Yuanye Biotechnology Co., Ltd (Shanghai, China), and omeprazole (Catalog No: H19990317) was purchased from AstraZeneca Pharmaceuticals (Wuxi, China).

### RE model

All the rats were fasted for 24 h without water. The rats were anesthetized by intraperitoneal injection of sodium pentobarbital at a dose of 40 mg/kg. The skin was prepared and a median epigastric incision of approximately 4 cm was made. To prevent the pyloric clip from moving distally, a 4.2 mm diameter pyloric clip with a double flat iron heart tie was prepared, placed at the pylorus and duodenum junction, and clamped. The closed end of the pyloric clip was tied with a 3/0 thread, and the other end was ligated to 2/3 of the fundus. Subsequently, the abdomen was stitched together layer by layer. Before closing the abdomen, saline (1–1.5 mL) was administered. After surgery, they drank water, fasted for 24 h, and were provided a standard pellet diet. After 3 days of model establishment, the groups were continuously tube-fed the above doses once daily for 14 days. Equal amounts of saline were administered through tube feeding to the sham and mod groups. Cefoperazone 0.1 g was administered intramuscularly for 3 days in a 0.1 mL/100 g volume to prevent infection.

### Cell culture

HET-1A cells were obtained from American Type Culture Collection and grown in T-75 flasks pre-coated with a mixture of 0.01 mg/ml fibronectin, 0.03 mg/ml vitrogen, and 0.01 mg/ml bovine serum albumin. These cells were maintained in BEBM supplemented with BEGM Single Quots, which includes 100 ng/ml EGF (Clonetics, Chicago, IL, USA), and fed every 2 day. The cells were seeded in 24-well plates that had already been pre-coated with a fibronectin-somatostatin combination. For every experiment, the cellular therapeutic doses of Brt were selected as 3.75, 7.5, and 15 μM as the low, medium, and high dose groups for the subsequent experiments, respectively.

### MTT assay

To derive viability/proliferation curves of long-term inhibitor-treated HET-1A cells, cells were seeded into a 96-well culture plate at a density 100 cells/well and grown in DMEM supplemented with 10% FBS. Cell viability was measured using MTT reagent (Sigma) dissolved in PBS (5 mg/ml). On the day of measurement, the medium was carefully replaced with fresh DMEM + 10% FBS with diluted MTT (1:10, 10% MTT), and incubated for 3,5 h at 37 °C. After removing the incubation medium, formazan crystals were dissolved in a 200 μl solution of DMSO. MTT reduction was quantified by measuring the light absorbance at 570 nm using the ELx800 absorbance microplate reader (BioTek Instruments, VT, USA). Each test was repeated at least four times in quadruplexes. The following calculation was performed to assess cell viability: cell viability (%) = (absorbance of the treated group/absorbance of the control group) × 100. This approach enabled the quantification of cell viability based on the relative absorbance levels between the treated and control groups.

### Esophageal injury score

The rat esophagus was dissected longitudinally and repeatedly rinsed with saline, and the mucous membrane of the esophagus was visualized, photographed, and scored as normal (0 points), mild (1 point), moderate (2 points), and severe (3 points) based on the degree of redness, erosion, fusion, or ulceration of the mucous membrane, and scored based on the different groups [29].

### Gastric content PH

Twelve hours before euthanasia, the rats underwent celiotomy, and the pylorus was ligated using a 3‐0 silk suture. Gastric juice was collected in a centrifuge tube and centrifuged at 3000 g for 10 min at 4 °C. The pH of the supernatant was measured using a pH meter (Mettler Toledo Delta 320).

### Acid and bile salt exposure in the HET-1A cells

The HET-1A cells were exposed to the acidic bile salt medium at PH 4.0. When the cells reached 75%-80% confluence, they were treated with media three times a day for 10 min each time [30].

### Hematoxylin–eosin staining (HE staining)

Waxed blocks of tissue chips were sliced, dried, and baked for 30 min. The stained sections were examined under a light microscope after being dewaxed in water, stained with hematoxylin, fractionated with acid alcohol, rinsed in running water, stained with eosin, dehydrated with a gradient of ethanol, cleared in xylene, and sealed with neutral resin.

### Toluidine blue stain

Dewaxing to water was performed first, xylene I for 5 min, xylene II for 5 min, xylene III for 5 min, anhydrous ethanol for 1 min, 95% ethanol for 1 min, 75% ethanol for 1 min, and rinsing with tap water for a few seconds. Drops of toluidine blue staining solution staining 20–30 min. Slightly wash with running water to remove excess staining solution. 95% ethanol color separation and control the color separation effect under the microscope. Final dehydration and transparency, anhydrous ethanol for 1 min, xylene 3 times for 1–2 min each time, neutral gum sealing.

### Cell grouping and transfection

HET-1A cells were divided into the mod, high-dose, si-TRIM31, and si-TRIM31 + high-dose groups. When the cell fusion reached 85%, TRIM31 siRNA was transfected into cells separately using Lipofectamine^®^ RNAi MAX transfection reagent, and cell sediment was collected 48 h after transfection to detect TRIM31 m RNA and protein expression levels was collected 48 h after transfection. The transfection reagent was purchased from Liji Biologicals (Product No.: AC04L092). The siRNA sequences were as follows:

**Table:** 

siTRIM31Sense (5ʹ—3ʹ)	AUGGAUCCUGCUGACAUCCAAUU
Antisense (5ʹ—3ʹ)	UUGGAUGUCAGCAGGAUCCAUUU

### Enzyme-linked immunosorbent assay (ELISA)

Assays were performed to measure IL-6, IL-1 β, and TNF-α in serum and cells using the kit.

### Immunohistochemical assay (IHC)

NLRP3 protein expression in the esophageal tissue was detected using IHC on paraffin-embedded sections. The sections were subjected to a series of steps, including dewaxing, rehydration, and blocking. These sections were then incubated with a primary antibody against a specific target: nucleotide-binding oligomerization domain-like NLRP3 (1:1000, 15,101, CST) overnight, and after phosphate-buffered saline (PBS) washing, sections for detection were incubated for 1 h with a secondary biotinylated antibody using the 3,3’-diaminobenzidine (DAB) substrate kit to visualize IHC staining and re-stained with hematoxylin. All the images were captured using a microscope. The fraction of collagen-rich or positive signal expression fields was determined using Image-Pro Plus software (Media Cybernetics).

### Western blot assay

Western blotting was performed to measure the expression levels of NLRP3, TRIM31, Claudin-4, Claudin-5, CaSR, ASC, C-Caspase-1, Caspase-1, and other proteins after different treatments. Sodium dodecyl sulfate–polyacrylamide gel electrophoresis (SDS-PAGE) was used to separate protein extracts from esophageal tissue or cell cultures before transferring them to PVDF membranes. The membranes were blocked, and primary antibodies against the target proteins were applied overnight: claudin-5 (1:1000, ab131259, Abcam), NLRP3 (1:1000, 15,101, CST), TRIM31 (1:1000, PA5-40,961, Invitrogen) claudin-4 (1. 1000, ab53156, Abcam), ASC (1:1000, 13,833, CST), Caspase-1 (1:1000, ab207802, Abcam), CaSR (1:1000, 73,303, CST), C-Caspase-1 (1:1000, 89,332, CST), and GAPDH (1:5000, 5174, CST) overnight at 4 °C. Membranes were washed with TBST and incubated with Anti-rabbit IgG (H + L) (1:5000, 14,708, CST) and Anti-mouse IgG (H + L) (1:5000, 14,709, CST) secondary antibodies for 2 h at room temperature. Gel imaging equipment was used to visualize the protein bands on the membrane, and ImageJ software was used to quantify them.

### Immunofluorescence (IF) assay

For IF analysis, the HET-1A cells after treatments were washed with PBS and were then blocked in 10% goat serum (#C0265, Beyotime Biotechnology) containing 0.3% Triton X-100 (#ST797, Beyotime Biotechnology) for 1 h at room temperature and incubated overnight with primary antibody NLRP3 (1:1000, 15,101, CST), TRIM31 (1:1000, PA5-40,961, Invitrogen) at 4 °C. The samples were then washed and incubated with secondary fluorescent antibodies, anti-rabbit IgG (H + L) (1:5000, 14,708, CST) and Anti-mouse IgG (H + L) (1:5000, 14,709, CST), for 45 min at room temperature in the dark. After washing, the samples were stained with 2-(4-aminophenyl)-6-indolinamidine dihydrochloride solution (DAPI; #C1006, Beyotime Biotechnology). Photographs were captured using a fluorescence microscope [31].

### Co-immunoprecipitation assay (Co-IP assay)

The interaction between TRIM31 and NLRP3 in HET-1A cells was examined using a Co-IP assay. Following treatment, IP buffer containing phosphatase and protease inhibitors was used to lyse the cells. Protein A/G agarose beads were used to pre-clear the cell lysates for 1 h at 4 °C. The supernatants were then incubated with anti-TRIM31(PA5-40,961; Invitrogen) antibody or normal IgG (sc-2027; Santa Cruz Biotechnology) as a negative control overnight at 4 °C, followed by protein A/G agarose beads for 2 h at 4 °C. The beads were boiled in loading buffer after washing with IP buffer. After washing off the non-specifically bound proteins, the remaining protein complexes were eluted from the beads and analyzed by protein blotting with NLRP3(1:1000, 15,101, CST), TRIM31 (1:1000, PA5-40,961, Invitrogen), or Ub ubiquitin antibodies (1:1000, 3936, CST).

### Molecular docking

Molecular docking experiments can predict complex shapes and binding affinities based on three-dimensional (3D) structures. Molecular docking combines these two techniques. A stiff 3D ligand was used to create a collection of conformations for sampling. The capacity of the method to investigate the ligand conformational space was assessed. The absolute value of the docking score represents the strength of binding capacity, visualized using the Dististory studio software.

### Statistical analysis

The data are displayed as the mean ± standard deviation (SD), after being evaluated with the GraphPad Prism 9.0 program. For comparisons involving three or more groups, one-way analysis of variance (ANOVA) with Bonferroni's correction was used to determine differences. *P* < 0.05 were considered statistically significant.

## Results

### Brt protects against RE-induced mucosal damage in rats

**Fig. 1:**
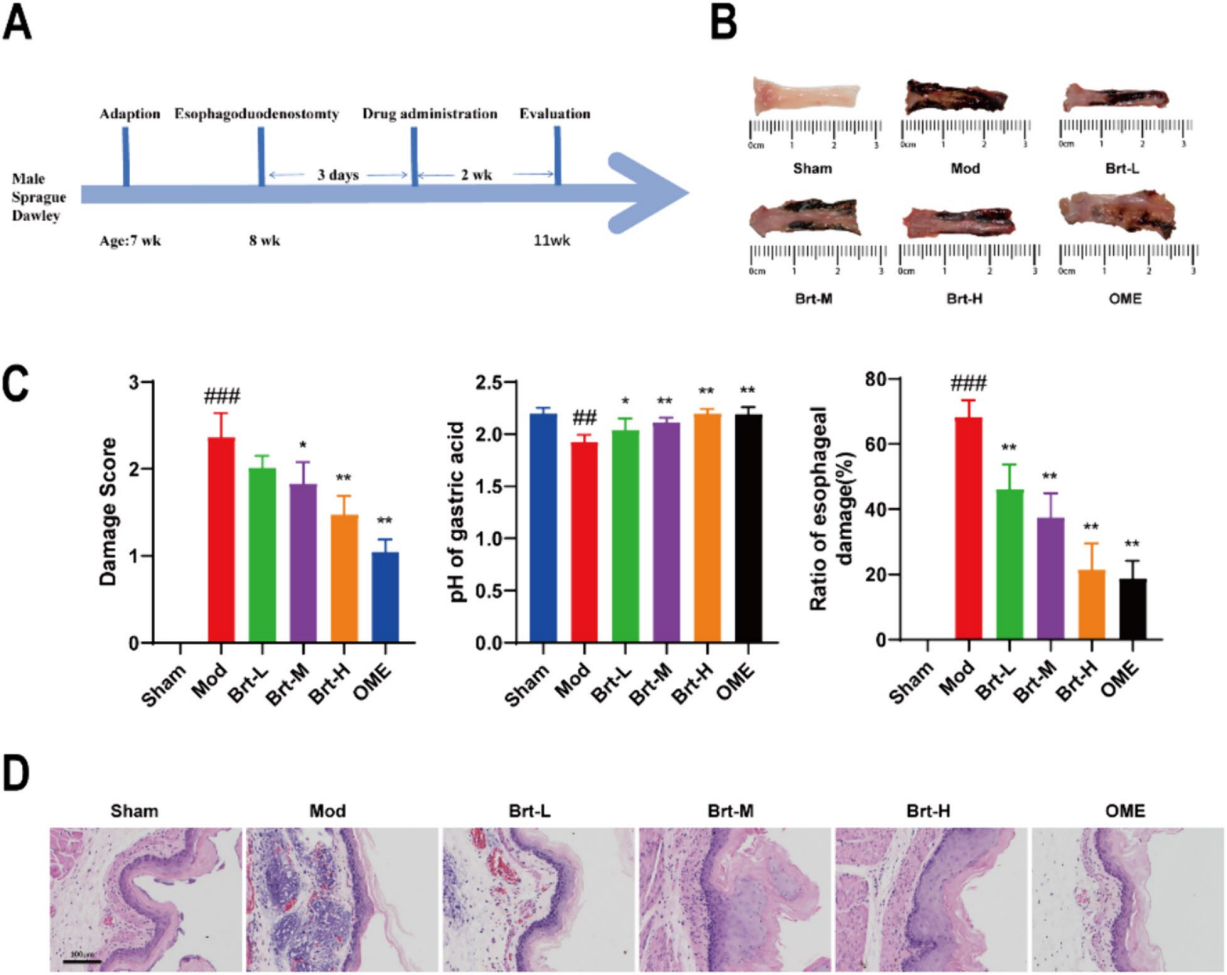
Effect of Brt on RE-induced mucosal damage. **A** Timeline of model establishment, grouping, and gastric administration in animal experiments; **B** Morphological examination using the esophagus in each group; **C** Esophageal injury score, Gastric acid pH of gastric contents in each group, and rate of esophageal injury in each group; **D** HE-stained esophageal tissue (n = 12 rats per group). Bar graphs represent the means ± SD. *P < 0 .05, **P < 0.01, compared with the mod group; #* P* < 0 .05 and ## *P* < 0.01, ###* P* < 0.001, compared with sham group

The procedure used for animal experiments is shown in Fig. 1A. The esophageal tissues of the different groups were scored according to Fig. S2. Morphological changes, such as hyperemia, congestion, and multiple erosions, were observed in rats with RE. (Fig. 1B). Compared with the sham group, the mod group showed extensive and severe redness and erosion, with a high rate of esophageal damage. With the oral administration of Brt, the rate of esophageal damage was lower (*P* < 0.01). As the dose increased, there was a coarser mucosa and significantly less redness and erosion. The OME group had a significant effect on punctate or striated redness and erosion, but no fusion, and the rate of esophageal damage was the lowest (*P* < 0.01). As administration increased, the score decreased gradually (*P* < 0.001) and was lower in the OME group than in the Brt-H group (*P* < 0.01). Gastric acid pH was significantly lower in the mod group than in the sham group (*P* < 0.01), higher in the Brt-L group than in the mod group (*P* < 0.05), and gradually increased after administration (Fig. 1C, P < 0.01).

HE staining observation showed that the esophageal mucosal structure was normal in the sham group. In the mod group, the esophageal mucosal mucosa was seen to have punctate vesicular erosion of mucosal tissues, squamous epithelial hyperplasia and thickening, flame cuticle hyperplasia, lamina propria limiting-like hyperplasia, and a large number of diffuse inflammatory cell infiltration were seen. Squamous epithelial hyperplasia, lamina propria elongation, and inflammatory cell infiltration of the esophageal mucosa were attenuated in both the Brt and OME groups compared to the mod group. (Fig. 1D). These data indicate that Brt elevates gastric acid pH and effectively improves RE-induced esophageal mucosal damage.

### Brt affects the Inflammatory factor and protein expressions of NLRP3 in esophageal tissues

**Fig. 2:**
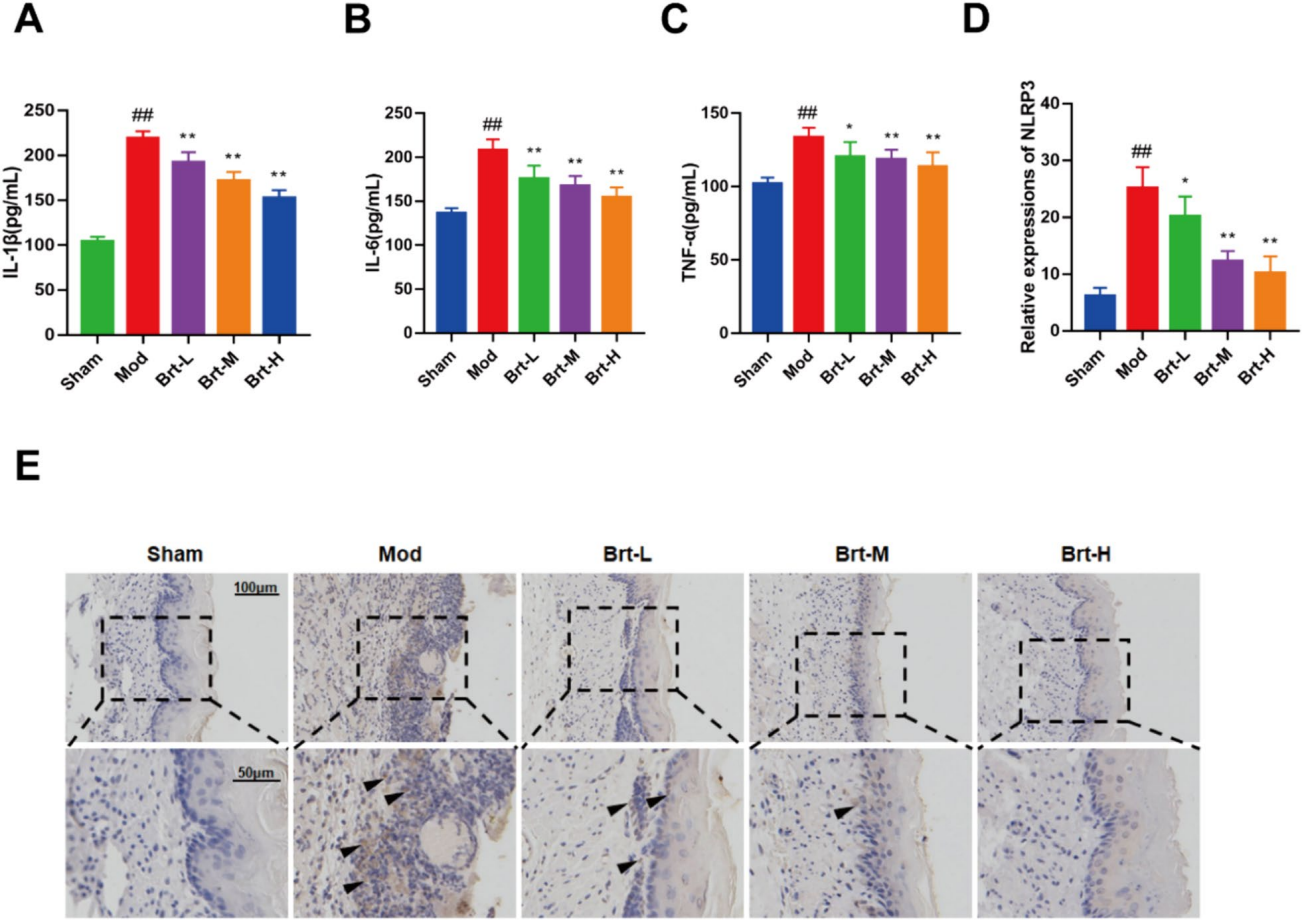
Brt reduces inflammatory factor levels and NLRP3 protein expression. **A**–**C** ELISA: serum IL-1 β, IL-6, and TNF-α results. **D–E** IHC assay of NLRP3 expression in esophageal tissues. (n = 6 rats per group). Bar graphs represent the means ± SD. *P < 0 .05 and **P < 0.01, compared with the mod group; #* P* < 0 .05 and ## *P* < 0.01, compared with sham group

The production of pro-inflammatory cytokines (TNF-α, IL-1 β, and IL-6) was evaluated using ELISA to examine the anti-inflammatory impact of Brt. In the RE rats, TNF-α, IL-1β, and IL-6 levels were noticeably greater than those of normal rat populations (*P* < 0.01). By administering at high and low doses of Brt to rats with esophagitis, the elevated levels were significantly reduced to levels close to those of the sham group (Fig. 2A–C, P < 0.01).

Next, IHC was used to evaluate the expression of NLRP3. The mod group had the highest relative expression of NLRP3 compared to the sham group (*P* < 0.01). The Brt-L group had a lower relative expression of NLRP3 than the mod group (*P* < 0.05). The trend of NLRP3 relative levels in the Brt-M and Brt-H groups was also gradually downregulated (Fig. 2D, E, P < 0.01). In conclusion, Brt reduced the levels of inflammatory factors and downregulated the protein expression of NLRP3.

The mod group had the highest relative expression of NLRP3 compared to the sham group (*P* < 0.01). The Brt-L group had a lower relative expression of NLRP3 compared to the mod group (*P* < 0.05). The trend of NLRP3 relative levels in the Brt-M and Brt-H groups was also gradually downregulated (Fig. 2D, E, P < 0.01). In conclusion, Brt reduced the level of Inflammatory factor and down-regulated the protein expression of NLRP3.

### Effect of Brt on TRIM31 and NLRP3 inflammatory vesicle levels and barrier function in esophageal tissues

**Fig. 3:**
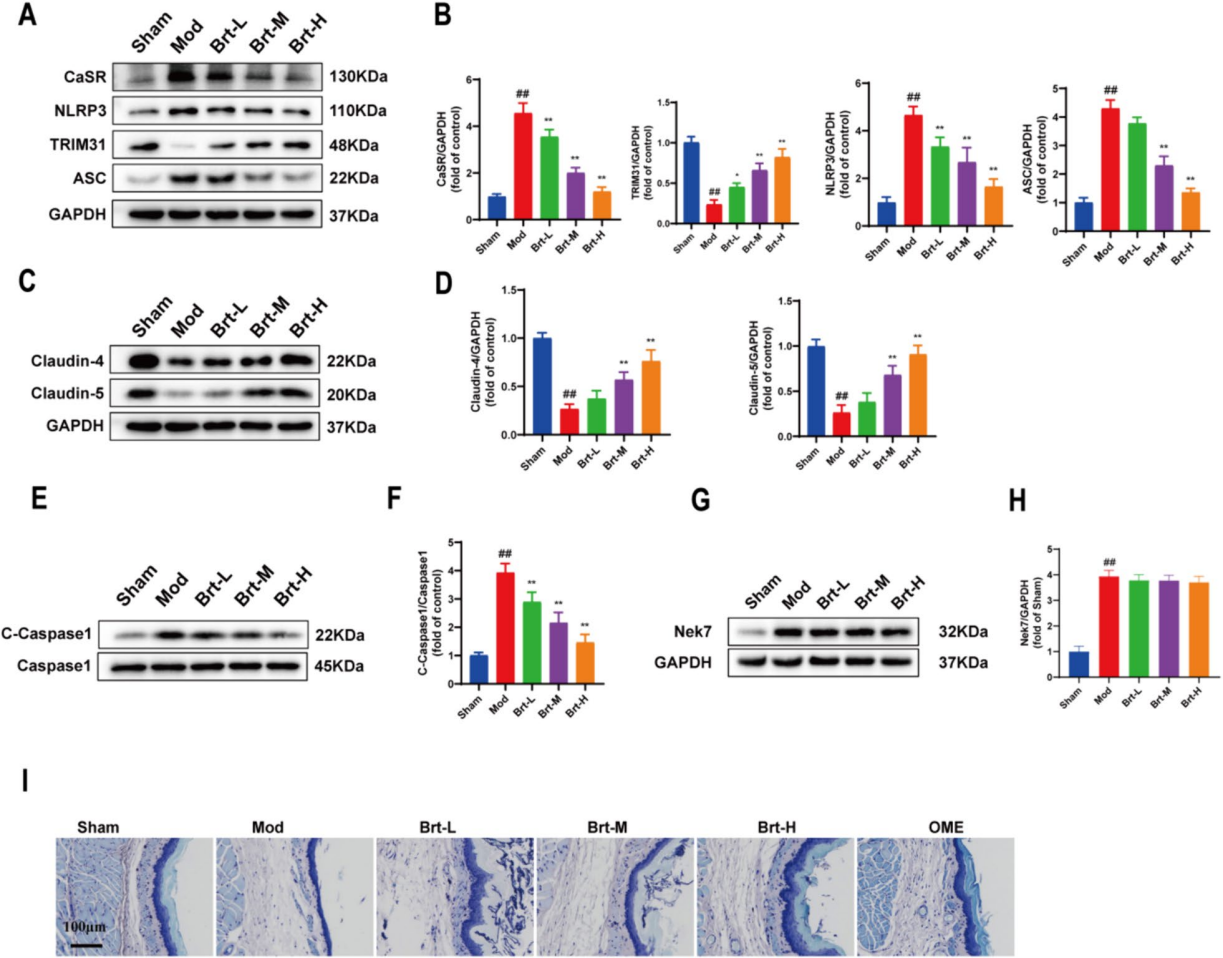
Brt can increase the expression of TRIM31 and reduce NLRP3 inflammatory factors in esophageal tissues. **A** Western blot assay. **B** The expression levels of CaSR, NLRP3, ASC, and TRIM31. **C**–**D** Western blotting assay of Claudin-4 and Claudin-5. **E**–**F** Western blotting analysis of C-Caspase-1/Caspase1 **G–H** Western blotting analysis of Nek7 **I** Toluidine blue stain (n = 3 rats per group). Bar graphs represent the means ± SD. *P < 0 .05 and **P < 0.01, compared with the mod group; # *P* < 0 .05 and ## *P* < 0.01, compared with sham group

To further determine the effect of Brt on RE, western blotting was used to assess the expression of pathway-related proteins. Because CaSR-mediated activation of NLRP3 in the esophageal tissue of modified RE rats leads to the secretion of pro-inflammatory cytokines, the following data were used to detect esophageal damage in RE rats. The protein levels of CaSR, NLRP3, C-Caspase1/Caspase-1, ASC, and Nek7 were significantly increased in the mod group than in the sham group (*P* < 0.01). Compared to the mod group, the protein levels of inflammatory factors in the administration group gradually decreased with increasing in the doses (Fig. 3A–B, E–H, P < 0.01). By contrast, TRIM31 was significantly down-regulated with the regulatory tight-linking proteins Claudin-4 and Claudin-5, and was gradually up-regulated after administration (*P* < 0.01). Claudin-4 and Claudin-5 are protein complexes that tightly connect neighboring cells and form a barrier that regulates each other to regulate the paracellular diffusion of ions. Compared to the mod group, the expression levels of Claudin-4 and Claudin-5 were increased, suggesting that Brt repaired the function of the esophageal epithelial barrier (Fig. 3C, D, P < 0.01). Toluidine blue staining revealed that the basal cells of the esophageal tissue in the mod group were fenestrated and disorganized, suggesting impaired protection of the esophageal epithelial barrier. Compared with the mod group, the basal cells of the esophageal tissue in the drug-delivered group were regularly arranged, and the epithelial tissue was intact, suggesting that the drug restored the barrier function of the esophageal epithelium (Fig. 3I).

In summary, these results suggested that Brt inhibited the activation of NLRP3 inflammasome and improved esophageal epithelial barrier function.

### Brt enables close interaction between TRIM31 and NLRP3 in esophageal tissues

**Fig. 4:**
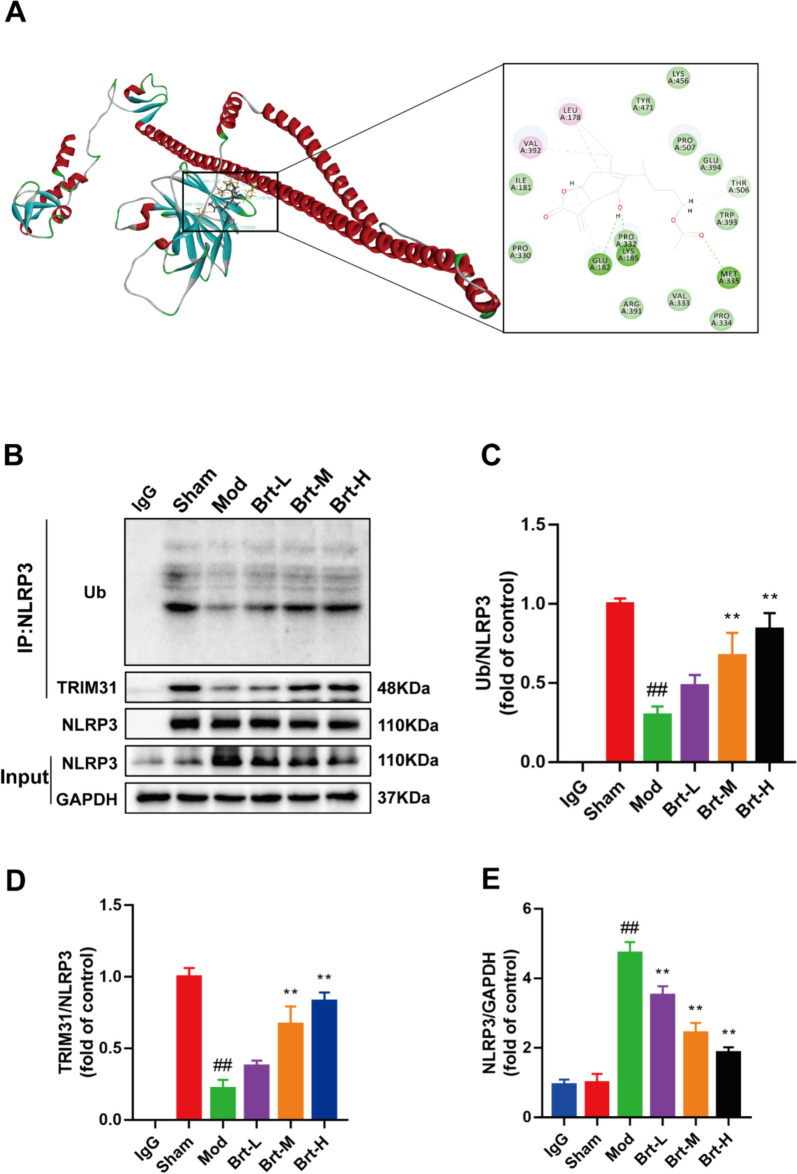
Brt increased NLRP3's ubiquitination and improved RE in esophageal tissues. **A** Molecular docking diagram of Brt and TRIM31. **B**–**E** Co-IP analysis of Ub/NLRP3, TRIM31, and NLRP3 (n = 3 rats per group). Data are shown as means ± SD. **P < 0.01, compared with the mod group; ##*P* < 0.01, compared with the sham group

Since the TRIM31 signaling pathway is one of the promising targets for the treatment of RE [21], Brt was effective in improving the treatment of RE in the previous findings, we hypothesized that Brt improves RE by promoting NLRP3 ubiquitination through TRIM31 in esophageal tissue. First, molecular docking was used to identify the binding pattern of Brt to TRIM31, and the docking score was used to assess the receptor-ligand binding capacity. The absolute value of the docking score represents the strength of binding capacity, visualized using Dististory studio software; the -CDOCKR energy score was 23.6618 kcal/mol, indicating good binding between Brt and the TRIM31 receptor with high binding energy. Pink represents hydrogen bonding, green represents alkyl bonding, and the hydrogen bonding interactions were GLU182, LYS185, and MET335(Fig. 4A).

Subsequently, we examined the effect of Brt on the binding of TRIM31 to NLRP3 using Co-IP. The levels of NLRP3 ubiquitination were lower in the mod group than in the sham group (*P* < 0.01), and higher in the Brt-M and Brt-H groups than in the mod group (Fig. 4C, P < 0.01). In the graph of the Co-IP results of TRIM31 and NLRP3, the binding levels of the two were the lowest in the mod group, compared to the sham group, however, integration levels became stronger as the dosage of the administered group increased (Fig. 4D). Brt promotes the binding of TRIM31 and NLRP3. Conversely, the protein levels of NLRP3 were elevated in the mod group compared to the sham group (*P* < 0.01) and downregulated in the Brt-L, Brt-M, and Brt-H groups in a decreasing trend compared to the mod group (Fig. 4E, P < 0.01). In summary, these data indicate that Brt improves the interaction between TRIM31 and NLRP3 in esophageal tissues.

### Brt inhibits the level of inflammatory factor in the acidic bile salts-induced HET-1A cells

**Fig. 5:**
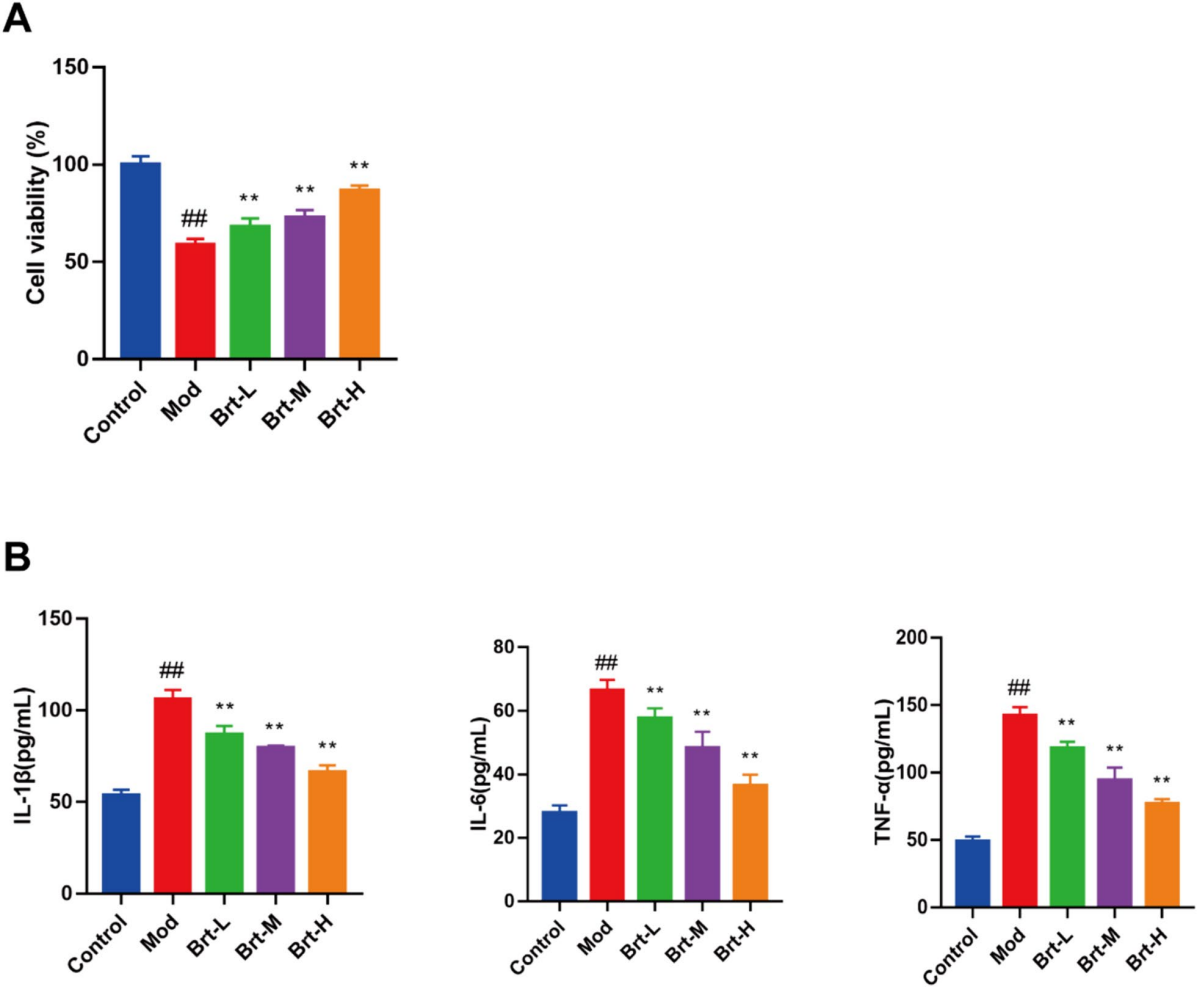
Brt successfully reduced inflammatory factors and enhanced HET-1A cell viability. **A** The MTT assay was used to measures cell viability. **B** ELISA was used to measure TNF-α, IL-1β, and IL-6 levels (n = 6 rats per group). Data are shown as means ± SD. **P < 0.01, compared with the mod group; ##*P* < 0.01, compared with the control group

To investigate whether the exposure of HET-1A monolayer cells to the acidic bile salt mixture affected the esophageal epithelium, the MTT assay was used to screen the administration concentrations and cellular activity of HET-1A cells in each group. The cellular activity was gradually down-regulated after the administration of Brt at the following concentrations: 3.75, 7.5, 15, 22.5, 30, 37.5, 75, 112.5 μM, and then used to select 3.75, 7.5, 15 μM was used as the low, medium and high dose groups for subsequent experiments to test cell viability, which was worse in the mod group compared with the control group (S1, *P* < 0.01), and with the increase of the administered dose, the cell viability of Brt-L, Brt-M, Brt-H was gradually up-regulated trend compared with the mod group (*P* < 0.01) (Fig. 5A, P < 0.01).

Next, esophageal epithelial cell line (HET-1A) inflammatory factor was detected using ELISA, and TNF-α, IL-1 β, and IL-6 levels in the mod group were all shown to be increased compared with those in the control group (*P* < 0.01). Inflammatory factors tended to decrease at low, medium, and high doses compared to those in the mod group (*P* < 0.01). Thus, Brt also inhibited and reduced the levels of proinflammatory factors in HET-1A cells (Fig. 5B). This suggests that Brt effectively increases acid bile salt-induced HET-1A cell viability and inhibits inflammatory factors.

### Brt regulates the protein expression of TRIM31/NLRP3 pathway in the acidic bile salts-induced HET-1A cells

**Fig. 6:**
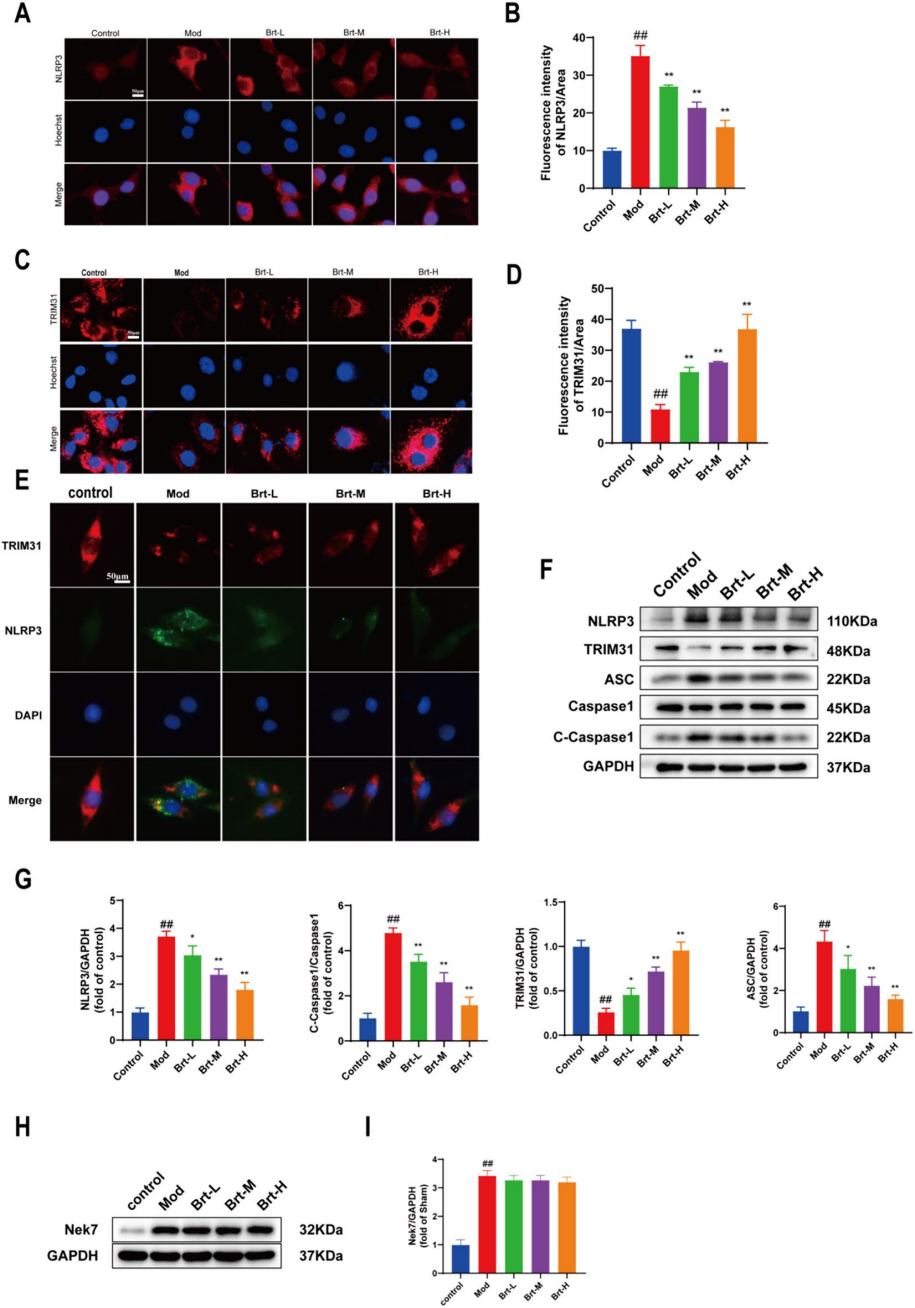
Brt stimulated the TRIM31/NLRP3 pathway in bile salt-induced HET-1A cells. **A**–**D** IF was used to measure TRIM31 and NLRP3 (n = 6 rats per group). **E** Co-localization of TRIM31 and NLRP3 **F-I** The Western blot analysis of TRIM31, ASC, NLRP3, C-Caspase1, and Nek7 (n = 3 rats per group). Data are shown as means ± SD. *P < 0.01, **P < 0.01, compared with the mod group; ##*P* < 0.01, compared with the control group

To further confirm the expression of the TRIM31 / NLRP3 inflammatory vesicle signaling pathway in the esophageal epithelium, we tested the IF of TRIM31/NLRP3 in HET-1A cells, and the mod group of NLRP3 had the highest intensity compared to the control group (*P* < 0.01). Compared with the mod group, the immunofluorescence intensity in the administered group decreased in a downward trend at higher doses (*P* < 0.01). By contrast, the TRIM31 immunofluorescence intensity was the lowest in the mod group than in the sham-operated group (*P* < 0.01). Compared to the mod group, the immunofluorescence intensity of the administered group tended to increase (Fig. 6A–D, P < 0.01). Co-localization analysis would enhance the presentation of the interaction between TRIM31 and NLRP3. The results of laser confocal microscopy showed that TRIM31 and NLRP3 co-localised, and the mod group had the minimum level of bonding compared with the sham group, while the Brt-H group had the highest highest level of bonding. With the increase of drug administration, the binding of TRIM31 and NLRP3 was also stronger. In conclusion, Brt can promote NLRP3 inflammasome attenuation through TRIM31 (Fig. 6E).

Furthermore, to assess the effect of bile salts and acid bile salts, the esophageal epithelium is damaged by mixed reflux, we studied the expression levels of TRIM31, ASC, and NLRP3, C-Caspase1, Nek7 in HET-1A cells stimulated by acid bile salt treatment, and compared to the control group, ASC, NLRP3, C-Caspase1, and Nek7 were higher in the mod group (*P* < 0.01) and lower in the mod group for TRIM31. Compared to the mod group, the protein expression levels of ASC, NLRP3, C-Caspase1, and Nek7 decreased (*P* < 0.01) and the protein expression levels of TRIM31 gradually increased as the dose in the administered group increased (Fig. 6F–I, P < 0.01). These data suggest that Brt weakens NLRP3 inflammasome activation in bile salt-induced HET-1A cells.

### Brt regulates inflammatory factor through TRIM31 and exacerbates cellular damage

**Fig. 7:**
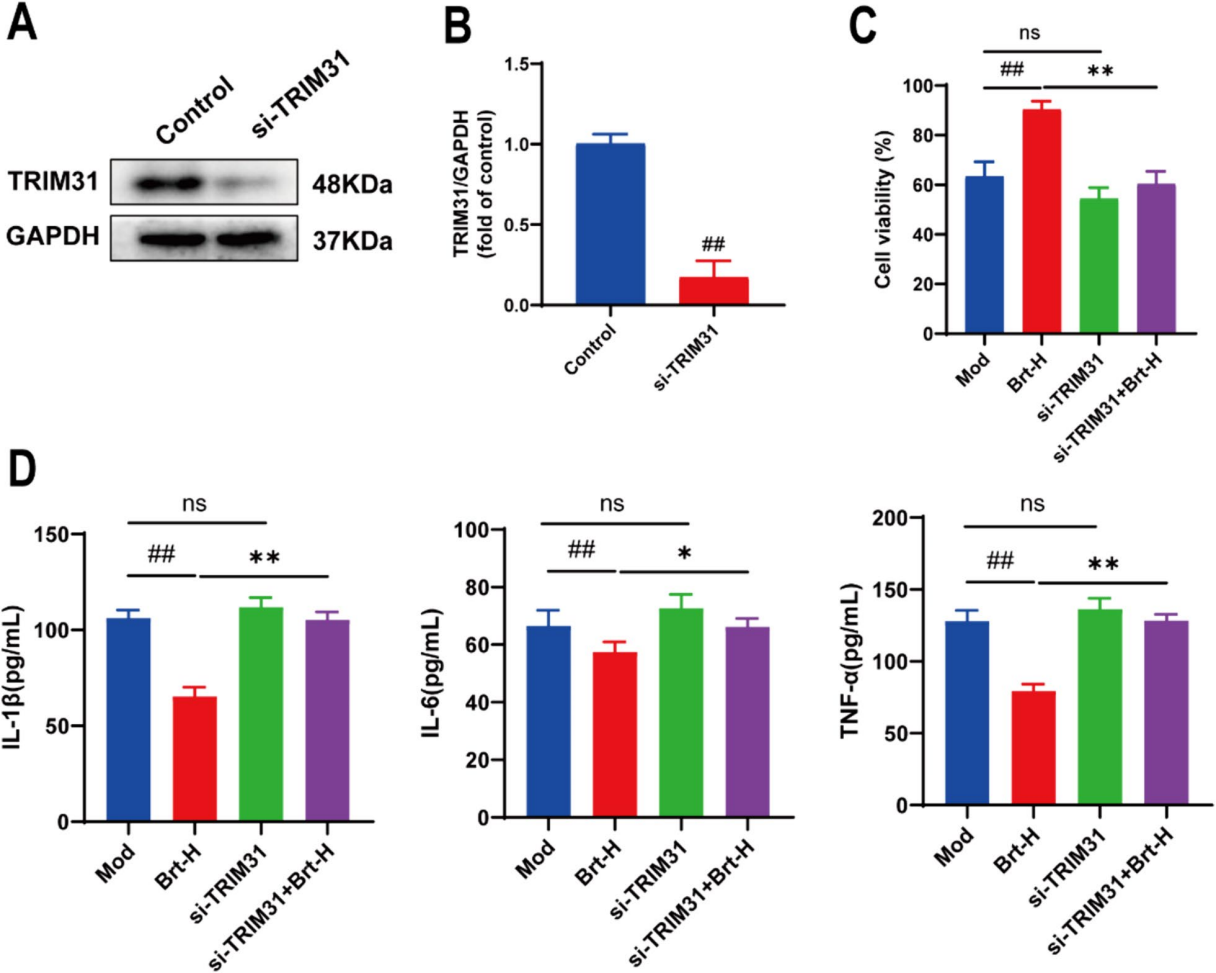
TRIM31 deficiency enhances inflammatory factor levels and increases cell damage **A**–**B** The Western blotting detected whether TRIM31 was successful in constructing the carrier. **C** The MTT test was used to detect cell activity in the mechanism group. **D** The Western blot expression levels of TNF-α, IL-1 β, and IL-6 were detected by ELISA (n = 3 rats per group). Data are shown as means ± SD. ##*P* < 0.01, compared with the mod group; and **P < 0.01, *P < 0.05 compared with Brt-H group

First, to gain a clearer understanding of the effect of Brt on protein expression levels of the si-TRIM31 vector, western blot analysis was performed. We observed that the expression level in the si-TRIM31 group was low compared to that in the control group (*P* < 0.01). It can be proven that the construction of the si-TRIM31 vector was correct, and the data support its validity (Fig. 7A–B).

Next, to investigate whether TRIM31 acts on Brt in bile salt-treated HET-1A cells, we established a mechanistic validation group that knocked down TRIM31 to construct the si-TRIM31 group. After grouping, we found that the cell viability was higher in the Brt-H group than in the mod group (*P* < 0.01). The viability of cells in the si-TRIM31 group was lower than that in the Brt-H group, whereas the viability of cells in the si-TRIM31 + Brt-H group was higher (Fig. 7C, P < 0.01).

Finally, ELISA was used again to detect inflammatory factors, the Brt-H group was shown to have lower expression levels of the pro-inflammatory cytokines TNF-α, IL-1 β, and IL-6 compared to the mod group (*P* < 0.01). In addition, the TNF-α, IL-1 β, I L-6 protein expression levels were higher in the si-TRIM31 + Brt-H group than in the Brt-H group (Fig. 7D, P < 0.05). No significant changes were observed in the si-TRIM31 group compared to the mod group (Fig. 7C–D). Thus, these data suggest that Brt-mediated TRIM31 is indeed effective in reducing inflammatory factor and HET-1A cell damage.

### TRIM31 silencing attenuates the effects of Brt on NLRP3 inflammasome

**Fig. 8:**
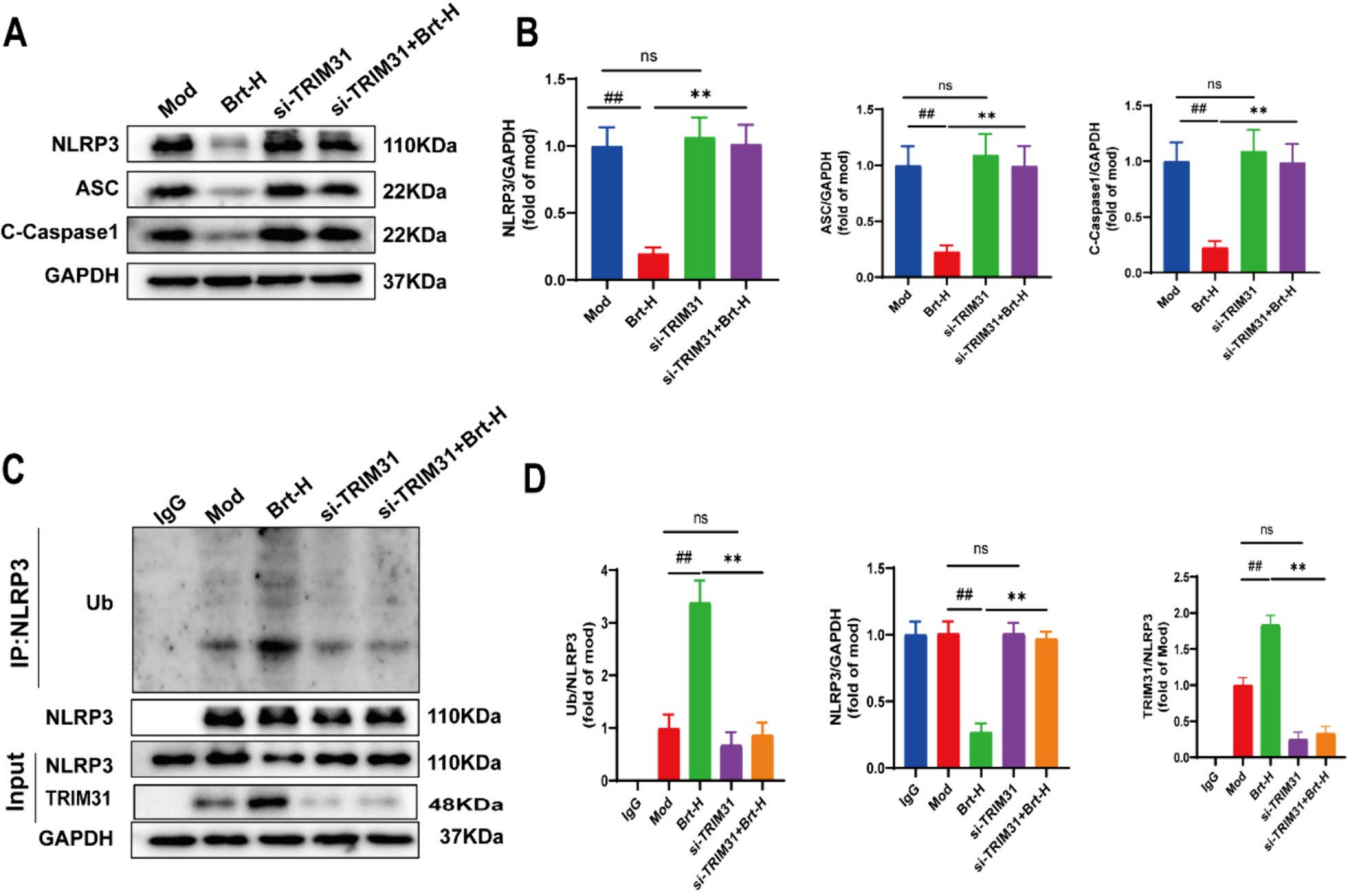
Absence of TRIM31 completely resolved the effect of Brt on NLRP3 ubiquitination. **A** and **B** Protein levels of ASC, NLRP3, and C-Caspase-1 were measured using western blotting. **C** and **D** Results of Co-IP assay of NLRP3, TRIM31, and Ub/NLRP3 (n = 3 rats per group). Data are shown as means ± SD. ##*P* < 0.01 compared with the mod group; and **P < 0.01, compared with Brt-H group

That TRIM31 may inhibit NLRP3 protein expression by promoting NLRP3 protein degradation [21]. After grouping, to further determine the effect of the si-TRIM31 vector on Brt action on RE bile salt-induced damage to rat esophageal HET-1A epithelial cells, the expression levels of the ASC and NLRP3, C-Caspase1 were measured using Western blotting. The protein expression levels of ASC, NLRP3, and C-Caspase1 were lower in the Brt-H group than in the mod group (*P* < 0.01). The protein expression levels of the si-TRIM31 + Brt-H group were higher than those of the Brt-H group (*P* < 0.01), and lower levels in the si-TRIM31 + Brt-H group than in the si-TRIM31 group (*P* < 0.01). This suggests that Brt does not reverse the alterations in factors such as NLRP3 after the knockdown of the gene TRIM31. (Fig. 8A, B).

Next, we performed IP to measure NLRP3 ubiquitination levels. Ub/NLRP3 protein expression was higher in the Brt-H group than in the mod group (*P* < 0.01) and lower in the si-TRIM31 + Brt-H and si-TRIM31 groups than in the Brt-H group (*P* < 0.01). The trend in NLRP3 expression in each group was the opposite to that described above. There were no significant changes in the si-TRIM31 group compared to the mod group. We can see that the expression of TRIM31 is lower in the mod group, so there is no significant difference between the si-TRIM31 group and the mod group (Fig. 8A–D). Thus, the knockdown of TRIM31 attenuated NLRP3 inflammatory vesicles in rats and almost eliminated the effect of Brt on NLRP3 ubiquitination (Fig. 8C, D) In conclusion, these data suggest that Brt might through TRIM31 to affect changes in NLRP3.

## Discussion

RE manifests as reflux of gastric and duodenal contents into the esophagus, causing inflammatory changes such as congestion, edema, and even erosion of the esophageal mucosa. Clinical manifestations include burning sensation and pain behind the sternum, acid reflux, heartburn, belching, pharyngeal obstruction, and gastric discomfort [32]. *Inula japonica Thunb.* was first used in the Divine Husbandman's Classic of the Materia Medica, and has a long history of medicinal use. According to the theory of traditional Chinese medicine, *Inula japonica Thunb.* has the effects of reducing qi, eliminating phlegm, dissolving water, and stopping vomiting. It is a common medicine used in the clinical treatment of wind-cold cough, phlegm, and fluid accumulation, chest and diaphragm congestion, wheezing and coughing up phlegm, vomiting, and stiff qi and heart of the lower part of the heart [33]. It is also the main component of Xuanfudaizhe Decoction (Treatise on Febrile Diseases), Xuanfuhua Decoction (Synopsis of Golden Chamber), and other classic prescriptions. It is commonly used to treat clinical gastroesophageal reflux, postoperative gastroparesis, functional dyspepsia, and other digestive system diseases [34, 35]. Brt is one of the main active components of *Inula japonica Thunb.,*with antitumor, anti-inflammatory, has other pharmacological activities [36]. In this study, Brt treatment improved the general condition and pathologic lesions of the esophageal epithelium in modified RE rats. In addition, Brt was effective in increasing the pH value of gastric acid in the lower esophagus. It has been shown that Claudins, a recently discovered family of multigene transmembrane integrins, are mainly responsible for tight junction permeability function [37]. By controlling the flow of proteins and lipids within the plasma membrane, they also play a key role in establishing and maintaining the polarity of epithelial cells and are a major barrier to the paracellular transport of solutes between neighboring cells [38, 39]. We investigated the effect of Brt on the function of the gastro-esophageal epithelial barrier using rats and found increased levels of Claudin-4 and Claudin-5 protein expression, a voucher for transmembrane proteins regulating their permeability and tightness.

Suppressing the level of inflammatory response has been an important goal in the treatment of patients with RE. NLRP3 inflammatory vesicles are closely associated with the pathogenesis of several diseases, including inflammatory bowel disease, gastric cancer and Hp gastritis [40–45]. The sensor molecule ASC and the effector protease Caspase 1 make up inflammatory vesicles, an intracellular supramolecular complex. Upon activation of the inflammatory vesicle sensor molecule, ASC self-associates into helical fibre assemblies that form ASC speckles or pyramids [46, 47], which serve as a molecular platform for neighborhood-induced autocatalytic activation [48]. In the absence of exogenous ATP, Ca^2+^ or other CaSR agonists activate NLRP3 inflammatory vesicles; however, CaSR knockdown inhibits inflammatory vesicle activation in response to a known NLRP3 activator [49]. Inflammatory vesicles are activated in response to a variety of cellular stresses and promote cysteine asparagine-dependent maturation of IL-1 β and IL-18 [citation needed]. NEK7, the smallest of the eleven (NEK1-NEK11) serine/threonine kinases of the mammalian NEK family, is expressed in a wide range of tissues, and its importance in the regulation of mitophagy and the inflammatory vesicle activation of NLRP3 has been well established. Its importance in mitotic regulation and NLRP3 inflammatory vesicle activation has been demonstrated [50], which involves direct binding of NEK7 to NLRP3. CaSR promotes epithelial cell differentiation and controls gastrointestinal immunity, and it is abundantly expressed in intact gastrointestinal polarised epithelial cells [51]. The primary effector mechanism of CaSR stimulation is the assembly and activation of NLRP3 inflammatory vesicles, followed by cysteine asparagine-1 activation, IL-1 β release, and apoptosis [52]. The results showed that Brt decreased NLRP3 activity, secretion of TNF-α, IL-1 β and IL-6 and altered the expression of CaSR, ASC, C-Caspase-1, Caspase-1, Nek7, and other inflammatory factors.

TRIM31 is a new member of the TRIM family that functions as an E3 ubiquitin ligase. TRIM31 is involved in various pathological events such as inflammatory diseases, protein quality control, autophagy, viral infections, and cancer development [53]. The TRIM family proteins are associated with the negative regulation of innate immune responses by facilitating the degradation of their respective substrates through the ubiquitin–proteasome pathway [19]. In addition, it has been shown that TRIM31 is also constitutively expressed in the intestine, restricting the expression of NLRP3 to relatively low levels, and therefore has a potential function in maintaining intestinal homeostasis [54]. In the present experimental study, it was found that Brt exerted a facilitating effect on TRIM31 compared to the model group, which was progressively increased with the administration of the drug. We hypothesized that the mechanism of action of TRIM31-mediated activation of NLRP3 inflammatory vesicles is an initiator of the inflammatory cascade response starting from the epithelium. We examined the binding capacity of TRIM31 to Brt by molecular docking and found a -CDOCKR_energy of 23.6618 kcal/moL, and that Brt binds tightly to the TRIM31 receptor. Ubiquitination is an important post-transcriptional modification that regulates the strength of the innate immune response, including modulation of the activity of the NLRP3 inflammasome [55]. We examined the interaction between TRIM31 and NLRP3 by CO-IP assay and found that Brt made the interaction between the two stronger and stronger, which in turn led to NLRP3 ubiquitination.

In vitro, we experimentally investigated the unique role of Brt as a modulator of RE therapy by acidic bile salt-driven HET-1A cells. It has been shown that TRIM31 promotes the degradation of protein pathways such as NLRP3 and reduces the therapeutic effect of downstream cytokines released in response to acidic bile salt stimulation [48]. In addition, TRIM31 also acts on NLRP3 simultaneously to ameliorate RE inflammatory damage. Results have also found that TRIM31 is a feedback inhibitor of NLRP3 inflammatory vesicle activity [56]. In this study, Brt increased cell viability in the administered group as detected by MTT, and ELISA assay revealed that Brt attenuated inflammatory factors such as TNF-α, IL-1 β and I L-6 secreted by NLRP3. Immunofluorescence co-localization analysis revealed that Brt enhanced the interaction between TRIM31 and NLRP3. Western Blot assay showed that Brt gradually decreased the expression of NLRP3 and enhanced the expression of TRIM31. It has been shown that TRIM31 is constitutively expressed in HET-1A cells and negatively regulates NLRP3 inflammasome activation to prevent unwanted activation in both resting and activated states. Manganet al. found that TRIM31 signaling activates the assembly of NLRP3-nucleated inflammatory vesicles, leading to cysteinyl asparagine 1-mediated IL-1 β cytokine family protein hydrolysis activation and induction of inflammatory cell apoptosis [57]. We subsequently validated the mechanism by using acidic bile salt-stimulated HET-1A cells to mimic mixed reflux injury in the esophageal epithelium and knocking down TRIM31 expression. TRIM31 knockdown was found to result in NLRP3 inflammatory vesicle activation, down-regulation of CaSR levels, reduced ASC release and cytokine secretion, counteracting the effects of Brt.

These data suggest that TRIM31 may be a useful therapeutic target to intervene in RE inflammasome activation, and TRIM31 promotes proteasomal degradation of NLRP3, which in turn inhibits the activation of NLRP3 inflammatory vesicles. In summary, Brt effectively promoted TRIM31-mediated ubiquitination of NLRP3 inflammatory vesicles and inhibited esophageal injury in modified RE rats.

## Conclusion

In the present study, we determined that Brt can promote TRIM31 attenuation of NLRP3 inflammasome-mediated RE. We verified that Brt can promote TRIM31 to inhibit inflammasome-mediated NLRP3 ubiquitination. Overall, these results revealed a novel mechanism of action for protecting Brt against RE and provided an experimental basis to lay the foundation for future clinical applications. In addition, the mechanism of the RE linkage between TRIM31 and NLRP3 requires further investigation. In conclusion, these findings suggest that Brt attenuates RE-mediated esophageal epithelial injury induced by acidic bile salt exposure by promoting NLRP3 ubiquitination through TRIM31.

### Supplementary Information

Supplementary material 1.

## Data Availability

All data generated or analyzed during this study are included in this published article and its supplementary.

## Abbreviations

RE: Reflux esophagitis
Brt: Britannilctone 1-O-acetate
GERD: Gastro-esophageal reflux disease
Caspase-1: Cysteine aspartate-specific protease-1
TRIM31: The tripartite motif contains 31
CaSR: The G-protein-coupled receptor calcium-sensitive receptor
MHC: Major histocompatibility complex
ASC: The adaptor apoptosis-associated speck-like protein containing a CARD
