# Supplementary material for: Britannilactone 1-O-acetate induced ubiquitination of NLRP3 inflammasome through TRIM31 as a protective mechanism against reflux esophagitis-induced esophageal injury
Source: Chin Med. 2024 Aug 30;19:118. doi: 10.1186/s13020-024-00986-y (PMC11363507; doi:10.1186/s13020-024-00986-y)
Supplement: Supplementary file 1 — Supplementary material 1. [file 13020_2024_986_MOESM1_ESM.docx]

S1


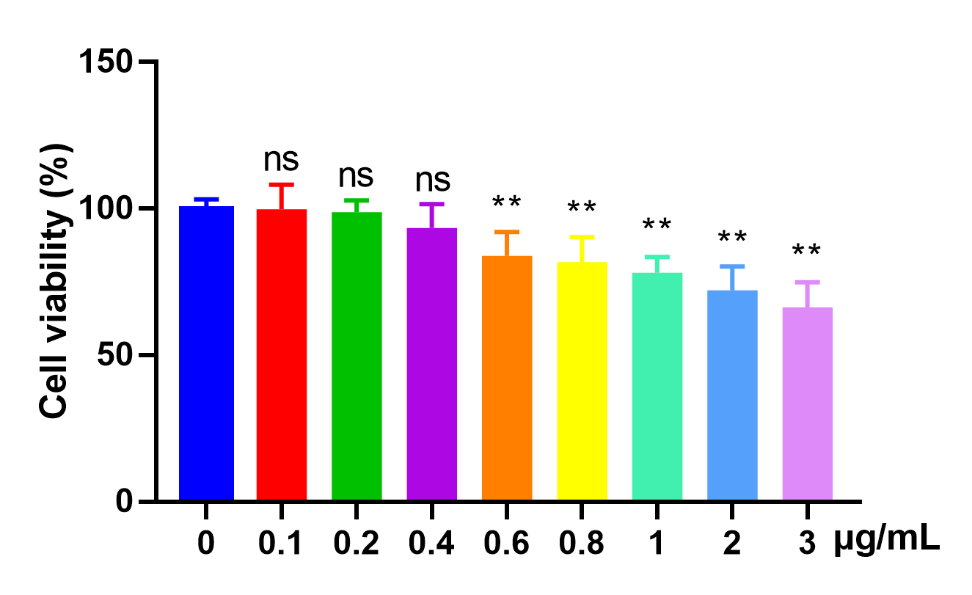


S2

| **Grading** | **Esophageal mucosal manifestations** | **Points** |
| --- | --- | --- |
| Class 0 | Normal (may have histological changes) | 0 |
| Class I | Redness and vesiculation in spots or stripes without fusion | 1 |
| Class II | There are streaks of redness, vesicles, and fusion, but not peripheral | 2 |
| Class III | Widespread lesions with redness, vesiculation that is peripheral, or ulceration | 3 |
